# Transcriptomic Analysis Reveals Functional Interaction of mRNA–lncRNA–miRNA in Steroidogenesis and Spermatogenesis of Gynogenetic Japanese Flounder (*Paralichthys olivaceus*)

**DOI:** 10.3390/biology11020213

**Published:** 2022-01-28

**Authors:** Jie Cheng, Fan Yang, Saisai Liu, Haitao Zhao, Wei Lu, Quanqi Zhang

**Affiliations:** 1 Key Laboratory of Marine Genetics and Breeding (Ocean University of China), Ministry of Education, 5 Yushan Road, Qingdao 266003, China; yangfan4303@stu.ouc.edu.cn (F.Y.); liusaisai97@163.com (S.L.); wyp13061301950@163.com (H.Z.); lw1981@ouc.edu.cn (W.L.); 2 Laboratory for Marine Fisheries Science and Food Production Processes, Pilot National Laboratory for Marine Science and Technology (Qingdao), 1 Wenhai Road, Qingdao 266237, China; 3 Key Laboratory of Tropical Aquatic Germplasm of Hainan Province, Sanya Oceanographic Institution, Ocean University of China, Sanya 572024, China

**Keywords:** *Paralichthys olivaceus*, gynogenesis, non-coding RNA, microRNA cluster, gametogenesis

## Abstract

**Simple Summary:**

Japanese flounder (*Paralichthys olivaceus*) is an important marine cultured fish that has a male heterogametic sex determination and shows significant sexual dimorphism with bigger females. Therefore, gynogenesis has presented great potential in breeding of all-female *P. olivaceus*. Non-coding RNAs are emerging as critical regulators of reproduction, and in this study, lncRNA–miRNA–mRNA interactions were investigated using high-throughput sequencing to reveal their roles in gynogenetic female and sex-reversed neo-male *P. olivaceus*. A considerable amount of steroidogenesis and spermatogenesis-related genes and ncRNAs were found, which may be regulated by *let-7*/*miR-125b* clusters and have significant functions during spermatogenesis of the neo-male *P. olivaceus*. These highly conserved and putatively interacted RNAs could replenish the genomic information of *P. olivaceus* and pave the way to understand the molecular mechanisms of gynogenesis effect and sex reversal in sexual development and gametogenesis of teleost fishes.

**Abstract:**

Teleost fishes exhibit extraordinary diversity, plasticity and adaptability with their sex determination and sexual development, and there is growing evidence that non-coding RNAs (ncRNAs) are emerging as critical regulators of reproduction. Japanese flounder (*Paralichthys olivaceus*) is an important marine cultured fish that presents significant sexual dimorphism with bigger females, in which gynogenesis has been applied for aquaculture industry. In order to reveal the regulatory mechanisms of sexual development in gynogenetic female and sex-reversed neo-male *P. olivaceus*, the lncRNA–miRNA–mRNA interactions were investigated using high-throughput sequencing. A total of 6772 differentially expressed mRNAs (DEmRNAs), 2284 DElncRNAs, and 244 DEmiRNAs were obtained between gynogenetic female ovaries and sex-reversed neo-male testes. Genes in the steroid hormone biosynthesis and secretion pathway were enriched and mostly significantly upregulated in neo-male testes. Subsequently, network analysis uncovered high functional specificity for gynogenetic *P. olivaceus* sperm motility, as co-expressed DEmRNAs were significantly enriched in microtubule and cytoskeleton-related biological processes. Clustered miRNAs were characterized in the *P. olivaceus* genome with examples of the largest conserved *let-7* clusters. The 20 *let-7* members are distributed in 11 clusters and may not transcribe together with their neighboring *miR-125b*, with *let-7* repressing *cyp11a* and *miR-125b* repressing *esr2b*, both as key steroidogenesis pathway genes. In summary, this study provides comprehensive insights into the mRNA–miRNA–lncRNA functional crosstalk in teleost sexual development and gametogenesis and will expand our understanding of ncRNA biology in teleost gynogenesis.

## 1. Introduction

Teleost fishes are the most abundant vertebrates on earth, and their sexual development exhibits significant diversity, plasticity, and adaptability with regard to sex determination and differentiation, spawning patterns, and mode of fertilization [1]. This is suggestive of teleost divergence at the level of molecular regulation. In vertebrate cells, the most transcribed RNAs are non-coding RNAs (ncRNAs), including microRNAs (miRNAs), long noncoding RNAs (lncRNAs), and *piwi*-interacting RNAs (piRNAs) et al., which have been extensively studied in the gene regulation of many biological processes such as immunology, development and cell proliferation, as well as sexual development and gametogenesis [2,3]. Specifically, mRNA translation could be subject to negative regulation by miRNAs, and the transcriptional stability of protein coding genes is also regulated by miRNAs [4]. Moreover, lncRNAs could be destabilized through the interaction with specific miRNAs, while lncRNAs can also compete with miRNA as sponges, with the reducing of miRNAs and rescuing of repressed target mRNAs [5,6]. Other lncRNAs can also produce miRNAs as their primary transcripts, leading to the repression of target mRNAs [5,7]. Therefore, these mRNA–miRNA–lncRNA regulatory patterns modulate gene expressions that drive almost all cellular processes central to vertebrate physiology and development.

Recently, the importance of gene regulation by ncRNAs and their targets is of increasing focus, and the vital role of mRNA–miRNA–lncRNA crosstalk in reproduction is well-evidenced in model organisms, indicating its involvement in different steps of fertilization, embryogenesis, sex determination and differentiation, gonadogenesis and gametogenesis [3,8], as well as covering germ cell specification, sex hormone responses, meiosis, placentation and non-genetic inheritance [9,10]. For example, the *miR-17-92* cluster and its paralogs have been extensively studied as being related to male germ cell development in both humans and mice [11,12]. Moreover, interactions between mRNAs and various ncRNAs also play essential roles in diverse reproductive processes, such as ovarian function [2,3] and hypothalamic–pituitary–gonadal (HPG) function [13], via acting as competing endogenous RNAs (ceRNAs). However, studies characterizing ncRNAs in reproduction of teleost species still lag far behind those for mammals despite teleosts accounting for half the diversity of vertebrate species. These studies are normally focused on certain ncRNA types (e.g., miRNAs), limiting functional interaction of different ncRNAs. For instance, many conserved miRNAs, such as *let-7*, *miR-202*, *miR-19*, *miR-430,* et al., represent regulated expressions during reproductive processes of several teleost species [14,15,16].

Japanese flounder (*Paralichthys olivaceus*) is an important marine cultured fish that is widely distributed along the coast of North Asia. It has a male heterogametic sex determination system with the influence of environmental factors such as water temperature and steroid hormones [17]. *P. olivaceus* exhibits significant sexual dimorphism in growth whereby females grow faster and bigger than males. Therefore, the generation of all-female *P. olivaceus* in aquaculture is of considerable advantage to increase production and economic profit [18]. Gynogenesis has been shown to be one of the established techniques to produce all female diploid offspring in aquatic animals, especially in genetic breeding and sex control of teleosts [19,20]. Although it has presented significant potential in genetic improvement and breeding of new *P. olivaceus* variations, information about the gonadal development and gametogenesis between gynogenetic XX females and sex-reversed XX neo-males is not well-understood [21,22]. Therefore, with the importance of ncRNAs in reproduction, here we report the comprehensive genome-wide characterization of mRNAs, lncRNAs, and miRNAs from gynogenetic *P. olivaceus* gonads and illustrate their expression patterns and functional interactions. All this information will pave the way for more insights into the regulatory crosstalk between mRNAs and ncRNAs during sexual development and gametogenesis in teleosts, and only then could it be employed to improve breeding and to produce a higher quality of commercially important aquaculture species.

## 2. Materials and Methods

### 2.1. Experiment Material and Sample Preparation

Gynogenetic diploid *P. olivaceus* were produced as previously described [23]. Briefly, parental *P. olivaceus* specimens from a commercial hatchery of Haiyang, China were cultured in seawater at 16 ± 1 °C until maturation. The fertilization of semen and eggs was checked before the experiment. The semen from one mature male was collected and diluted 100 times with Ringer’s solution (without Ca^2+^ and Mg^2+^) at 0–2 °C without activating the sperm motility and was immediately irradiated under ultraviolet light of wavelength 254 nm at 112 mJ/cm^2^ for 40 s. Then the semen was inseminated into eggs from one mature female for 2 min of fertilization, and the eggs were used to induce a meiogynogenetic diploid by hydrostatic pressure of 60 MPa for 6 min to inhibit second polar body extrusion. The fertilized eggs were incubated under 16 ± 1 °C. The proportion of sex-reversed neo-males in the gynogenetic progeny was increased by high temperature (28 °C) acclimation during the critical period of *P. olivaceus* sex differentiation (50–120 dpf). The induced gynogenetic *P. olivaceus* specimens were further raised for 1.5 years, and afterwards, six 1.5-year-old gynogenetic *P. olivaceus* individuals (three females and three neo-males) were sampled, and their gonads were collected and frozen using liquid nitrogen and stored at −80 °C for further usage.

### 2.2. RNA Extraction, Library Construction and Sequencing

Total RNA was extracted from the gonads with Trizol Reagent (Invitrogen, Carlsbad, CA, USA) and treated by RNase-free DNase I (TaKaRa, Dalian, China) to eliminate genomic DNA. The quantity and quality of total RNA were examined with 1.5% agarose gel electrophoresis and spectrophotometry by NanoPhotometer Pearl 360 (Implen, Munich, Germany). For sequencing library construction, rRNA was removed from total RNA using the Ribo-Zero^TM^ rRNA removal Kit (Epicentre, Madison, WI, USA). An Agilent 2100 Bioanalyzer (Agilent Technologies, Santa Clara, CA, USA) was employed to measure the quantity and quality of retained RNA without rRNA. Six libraries (three testes and three ovaries) were constructed with NEBNext Ultra^TM^ II Directional RNA Library Prep Kit for Illumina (NEB, Ipswich, MA, USA) for mRNA and lncRNA sequencing. In addition, six small RNA libraries from the same individuals (three testes and three ovaries) were constructed using TruSeq Small RNA sample Prep Kit (Illumina, San Diego, CA, USA). These libraries were run on the Illumina HiSeq 2500 sequencer (Illumina, San Diego, CA, USA) with PE 150 and SE50 for lncRNA and miRNA, respectively.

### 2.3. Transcriptome Analysis and lncRNA Characterization

The raw reads from RNA-seq were adaptor-trimmed and quality-filtered. Each RNA-seq dataset was aligned to the *P. olivaceus* genome (Flounder_ref_guided_V1.0, PRJNA344006) using Hisat2 (-rna-strandness RF), and the transcripts were assembled using the StringTie pipeline [24,25]. The abundance of transcripts was quantified with transcripts per million reads (TPM). With the merged results, the remaining transcripts were applied to identify lncRNAs with the following criteria: (i) transcripts with the length > 200 nucleotides (nts) and exon number > 2; (ii) the transcript coding potential was estimated using the coding potential calculator (CPC) [26] with CPC scores < 0 and coding non-coding index (CNCI) analysis with CNCI score < 0 [27]; (iii) the transcript coding potential was further estimated against the Pfam database with transcript length > 200 nts, *E*-value < 0.001, and no ORF > 100 amino acids, and the coding probability from coding potential assessment tool (CPAT) was < 0.85 [28]; (iv) transcripts were also examined against Nr, the Kyoto Encyclopedia of Genes and Genomes (KEGG), and the Swiss-Protein database (Swiss-Prot) by BLASTX (E-value < 1 × 10^−10^, coverage > 85%, and identity > 95%) to retain transcripts without significant homology to known proteins.

### 2.4. Differential Expression of lncRNA and mRNA with Functional Annotation

Differentially expressed mRNAs (DEmRNAs) and lncRNAs (DElncRNAs) between testes and ovaries were identified using DESeq2 [29]. Any mRNA or lncRNA with log_2_|fold change| > 2 and adjusted *p*-value < 0.001 were identified as DEmRNAs or DElncRNAs. The putative cis- and trans- targets of lncRNAs were predicted. With cis- regulation, lncRNAs target neighboring genes about 100 kb up- and down- stream. Trans- regulation refers to the lncRNAs acting on other genes with Pearson correlation coefficients (PCC) (|r| > 0.95) between the expression levels of lncRNAs and mRNAs. Then, functional annotation was conducted with Gene Ontology (GO) and KEGG for the DEmRNAs and target genes of DElncRNAs using Omicshare tools (https://www.omicshare.com/, accessed on 22 August 2021).

### 2.5. miRNA Identification, Differential Expression and Functional Annotation

Raw reads from small RNA-seq were processed to remove adapters, low complexity, common RNA families (rRNA, tRNA, snRNA and snoRNA), and repeats. Unique sequences with lengths between 18–28 nts were mapped to miRNA precursors of miRBase 21.0 (http://www.mirbase.org/, accessed on 12 March 2021) by BLAST to identify known and novel miRNAs. Length variation at both the 3′ and 5′ ends and one mismatch inside the sequence were accepted in the alignment. The unique sequences were mapped to selected species precursors and mature miRNAs in miRBase by BLAST, considered as known miRNAs, and the mapped pre-miRNAs were further mapped using BLAST against the *P. olivaceus* genome to obtain their genomic locations. The unmapped sequences were subjected to BLAST against other selected genomes, and the RNA hairpin structures were predicated with the flanking 80 nt sequences by RNAfold (http://rna.tbi.univie.ac.at/cgi-bin/RNAfold.cgi, accessed on 27 December 2020). The frequency of the miRNAs was normalized to TPM in each sample. Differentially expressed miRNAs (DEmiRNAs) were defined by log_2_|fold change| > 1 and adjusted *p*-value < 0.05. The transcripts and identified miRNAs were further submitted to TargetScan [30] (context score percentile > 50) and miRanda [31] (Max energy < −10) to predict their target genes.

### 2.6. Construction of DEmRNA–DEmiRNA–DElncRNA Network

To investigate the interactions of mRNAs and ncRNAs, networks were constructed based on the complementary pairs of miRNA–lncRNA and miRNA–mRNA. The DElncRNA–DEmiRNA–DEmRNA network was constructed as follows: First, target DEmRNAs or DElncRNAs of the DEmiRNAs were predicted by miRanda, and expression correlation between DElncRNAs and DEmRNAs that shared the same DEmiRNA was examined through PCC. The lncRNA–mRNA were considered as co-expression pairs with a threshold PCC > 0.9 and *p* < 0.05. Then, the lncRNA–mRNA pairs, as common miRNA targets, were obtained as co-expression competing triplets. Finally, all these co-expression competing triplets were employed for lncRNA–miRNA–mRNA network construction and were visualized with Cytoscape [32].

### 2.7. miRNA Cluster Identification

Clustered miRNAs were identified as colocalized within 10 Kb on the *P. olivaceus* genome. The information of precursors, mature miRNAs and genomic location of selected vertebrates were obtained from miRBase 21.0. The homolog sequences and genomic locations of *let-7* members were obtained in selected vertebrates. The consensus sequence of *let-7* in *P. olivaceus* was illustrated using weblogo (http://weblogo.berkeley.edu/, accessed on 6 January 2021). Precursors of *let-7* clusters and the paralogous groups were aligned with Clustal X by multiple alignments, and the maximum likelihood (ML) phylogenetic trees based on miRNAs were constructed with MEGA 7 [33] using a bootstrap of 1000 replications.

### 2.8. qRT-PCR Analysis

The RNA extracted from gonads were reverse transcribed using the All-In-One 5× RT MasterMix (abm, Vancouver, CA, Canada) for mRNA/lncRNA and the Mir-X™ miRNA First-Strand Synthesis Kit (Clontech, Mountain View, CA, USA) for miRNA described as per the manufacture’s protocol. qRT-PCR was conducted in a 20 µL system including a 2 μL template (5 ng/μL), 0.4 μL of each primer (10 μM), 10 μL 2× SYBR Green qPCR Master Mix (US Everbright Inc., Suzhou, Jiangsu, China), and 7.2 μL nuclease-free water through a Roche LightCycler^®^ 480 (Roche, Mannheim, Germany). The primers used for qRT-PCR are shown in Table S1a. The qRT-PCR reaction was 95 °C (5 min) for initial denaturation, followed by 40 cycles at 95 °C (15 s), 60 °C (45s for mRNA/lncRNA and 20s for miRNA) and 72 °C (15 s), and a final extension at 72 °C for 10 min. The reaction of each sample was conducted in triplicate. Melting curves were evaluated after amplification to identify the amplicon specificity. *β-actin* and *ubiquitin-conjugating enzyme* (*UBCE*) were combined as the best reference genes to normalize the relative expressions of mRNA and lncRNA [34] while *miR-22-3p* and *miR-23a-3p* were applied as the best combined references to determine miRNA relative expression [35]. Target gene expression was calculated with the 2^−ΔCt^ method, and the statistical analysis was performed using SPSS 20.0 with *p* value ≤ 0.05 to be the significant difference.

### 2.9. Vector Construction and Dual-Luciferase Activity Assay

The 3′-UTR fragments of *cyp11a*, *hsd3b1* and *esr2b* containing putative seed regions of miRNAs (*let-7* and *miR-125b*) were amplified from the *P. olivaceus* gonad cDNA or DNA templates, respectively. All the primers are present in Table S1b. The PCR products were sequenced for confirmation. All gel purified fragments were digested by double enzymes, then ligated into the pmirGLO dual-luciferase miRNA target expression vector (Promega, Madison, WI, USA) at the 3′ end of firefly luciferase (luc2) gene and further confirmed by DNA sequencing. The human HeLa cell line was culture with Dulbecco modified Eagle medium (Biological Industries, Beit-Haemek, Israel), 10% fetal bovine serum (Gibco, GrandIsland, NY, USA), 1% penicillin-streptomycin (Biological Industries), and 1% MEM non-essential amino acids (Biological Industries) at 37 °C with 5% CO_2_. For the luciferase reporter assay, HeLa cells were seeded in 24-well plates and transfected with 20 pmol miRNA mimics (*let-7* and *miR-125b*) or negative controls (NC) (sense: 5′-UUCUCCGAACGUGUCACGUTT-3′ and antisense: 5′-ACGUGACACGUUCGGAGAATT-3′) together with 500 ng luciferase reporter plasmids containing specific seed regions of putative target genes using the Lipofectamine™ 3000 transfection reagent. At 48 h post transfection, cells were collected for luciferase activity detection using the Dual-Luciferase Reporter Assay System (E1910, Promega, Madison, WI, USA). Luciferase signals were determined by Synergy^TM^ H1 (BioTek, Winooski, VT, USA), and the relative luciferase activities were calculated as the signal ratio between firefly luciferase (luc2) readouts and renilla luciferase (luc1) readouts with four independent transfection experiments.

## 3. Results and Discussion

### 3.1. Transcriptome Profiles of Gynogenetic P. olivaceus Gonads

**Figure 1:**
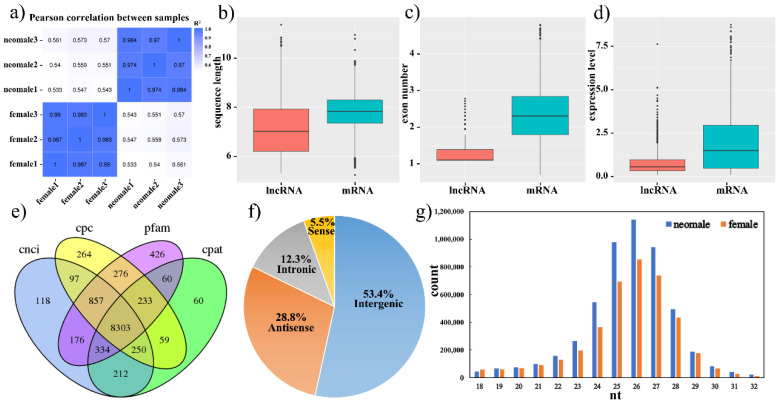
Characterization of mRNAs, lncRNAs and miRNAs in ovary and testis of gynogenetic *P. olivaceus*. (**a**) The Pearson coefficient from TPM values of gonad samples; (**b**) transcript length, (**c**) exon number, and (**d**) expression level between lncRNAs and mRNAs; (**e**) Venn diagram of lncRNAs identified with four methods; (**f**) the classification of lncRNAs; (**g**) length distribution of miRNAs.

Three testes and three ovaries were sampled from 1.5-year-old gynogenetic *P. olivaceus* for RNA-seq and small RNA-seq analysis. A total of 630,880,834 raw pair-end reads and 617,434,394 clean reads were generated from the six strand-specific RNA-seq (ssRNA-seq) libraries (Figure 1a and Table S2a). After mapping the ssRNA-seq clean reads to the *P. olivaceus* genome (Flounder_ref_guided_V1.0), a total of 22,911 mRNAs were detected and 8303 lncRNAs were de novo predicted. We compared the general characteristics of the putative lncRNAs with mRNAs, which revealed that lncRNAs had shorter transcripts than mRNAs, as well as fewer exons and lower expression levels than mRNAs (Figure 1b–d and Table S3a,b). For instance, the identified lncRNAs (median 1140 bp; average 2651.7 bp) were shorter than the mRNAs (median 2501 bp; average 3057.9 bp) (Figure 1b). A high percentage (96.47%) of lncRNAs had 2–4 exons (Figure 1c), and the exon counts of the putative lncRNAs (average 2.4 exons) were less than those of mRNAs (average 12.1 exons) (Figure 1c). Moreover, a higher percentage (60.30%) of lncRNAs had low expression (FPKM < 1) than those of mRNAs (31.40%) (Figure 1d and Table S3a,b), which indicated that lncRNAs had different expression patterns from protein-coding genes in *P. olivaceus*.

Moreover, the obtained 8303 lncRNAs from integrating identification methods (Figure 1e) were further categorized as four types: intergenic lncRNAs (lincRNAs), sense and anti-sense lncRNAs, and intronic lncRNAs, according to their genomic locations to protein-coding genes. Most of the lncRNAs (4437, 53.4%) were distributed in intergenic regions and only 1022 (12.3%) and 2388 (28.8%) lncRNAs were intronic and antisense lncRNAs of protein-coding genes, respectively (Figure 1f). In addition, for the small RNA-seq analysis, a total of 99,889,413 clean reads were obtained from 181,802,212 raw reads generated in the six miRNA libraries (Table S2b). After length filter and annotation, about 50% clean reads ranged 20–28 nt in length (Figure 1g) and could be classified as 824 miRNAs, including 593 known miRNAs and 231 novel miRNAs.

### 3.2. Differential Expression of mRNAs, lncRNAs and miRNAs in Gynogenetic P. olivaceus Gonads

**Figure 2:**
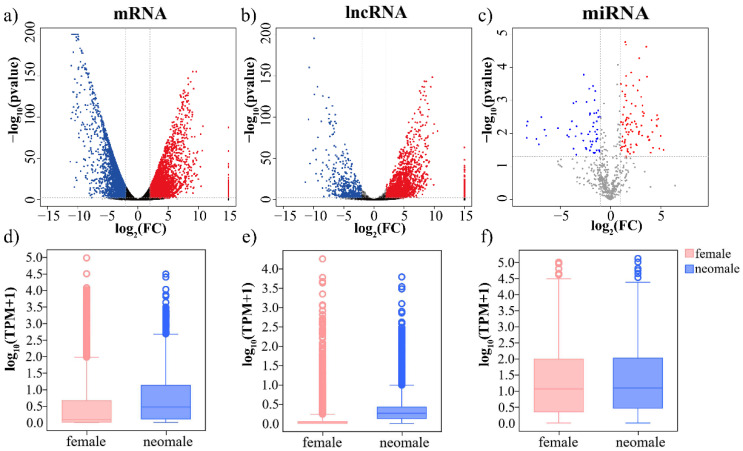
Differential expression of mRNAs (**a**,**d**), lncRNAs (**b**,**e**) and miRNAs (**c**,**f**) in gynogenetic *P. olivaceus* gonads. Red spots indicate up-regulation in testes, and blue spots represent down-regulation in testes.

Expression profiles of mRNA and lncRNA were investigated, and a total of 6772 DEmRNAs (3541 testis-biased and 3231 ovary-biased) were identified in gonads of gynogenetic *P. olivaceus* (Figure 2a and Table 1). For ncRNAs, 2284 lncRNAs (1870 testis-biased and 414 ovary-biased, Figure 2b) and 244 miRNAs (146 testis-biased and 98 ovary-biased, Figure 2c) were differentially expressed between testes and ovaries, respectively (Table 1 and Table S3a,b). There were a higher proportion of testis-biased lncRNAs (22.5%) and miRNAs (17.7%) than mRNAs (15.5%) (Table 1), suggesting more active functions of ncRNA in the neo-male testis. Moreover, the expression levels of mRNAs and lncRNAs were both higher in testes than in ovaries (Figure 2d,e and Table S3a,b), while not much difference in miRNA was obtained between testes and ovaries (Figure 2f and Table S3c).

**Figure 3:**
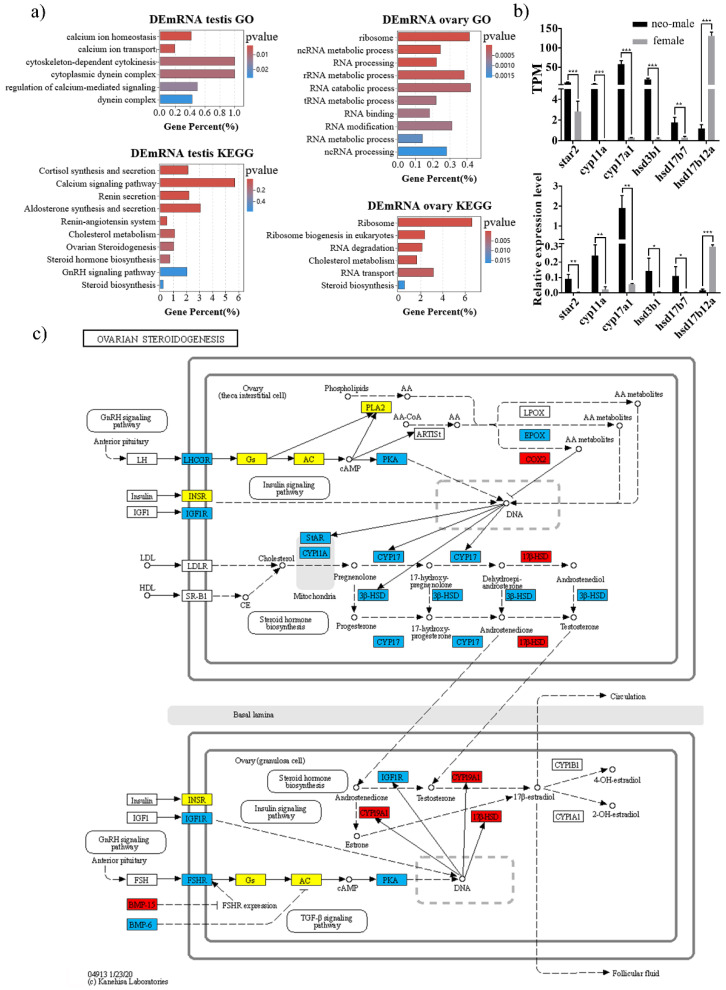
Functional annotation of DEmRNAs with steroid hormone biosynthesis pathway in *P. olivaceus*. (**a**) GO and KEGG enrichment of testis- and ovary-biased DEmRNAs; (**b**) differential expression of selected genes in ovarian steroidogenesis pathway by RNA-seq and qRT-PCR; (**c**) DEmRNAs in the steroidogenesis pathway. Blue boxes represent testis-biased genes, red boxes represent ovary-biased genes, and yellow boxes represent genes expressed in both testis and ovary. Data are shown as mean ± SD (*n* = 3). Asterisks indicate statistical significance between groups (* *p* < 0.05; ** *p* < 0.01; *** *p* < 0.001).

GO and KEGG annotation were employed to determine the biological significance of DEmRNAs (Table S4). As a result, testis-biased DEmRNAs were enriched in reproduction-related functions including GO terms “calcium ion homeostasis”, “cytoplasmic dynein complex”, “dynein complex” and KEGG pathways “cortisol synthesis and secretion” and “calcium signaling pathway” (Figure 3a and Table S4a,b), which were mostly related to functional sperm development. For example, calcium is necessary for a multitude of sperm involving processes, ranging from regulation of steroidogenesis at spermatogenesis, sperm motility, capacitation, and acrosome reaction before fertilization [36]. Moreover, ovary-biased DEmRNAs were mostly enriched in “ribosome”, “ncRNA metabolic process”, “rRNA metabolic process”, “tRNA metabolic process”, “ncRNA processing” and “steroid biosynthesis”, which may indicate vigorous RNA and protein metabolic activities in ovaries (Figure 3a and Table S4c,d).

To investigate the function of DElncRNAs, the cis- and trans- targets of lncRNAs were predicted. For the cis-regulation, prediction revealed 7283 and 1846 potential target genes for 1450 up- and 303 down-regulated DElncRNAs, respectively. GO and KEGG annotation showed that cis-targets of up-DElncRNAs were enriched in “fertilization” and “steroid biosynthesis” (Table S5a,b), while cis-targets of down-DElncRNAs were represented in “steroid binding”, “microtubule binding”, “sex differentiation”, “gonad development” and “MAPK signaling pathway” (Table S5c,d). On the other side, the trans-regulation of DElncRNA were identified based on their expression correlation coefficients (PCC > 0.95) with mRNAs. A total of 1903 and 86 protein-coding genes were detected in trans with 1102 up- and 83 down-regulated DElncRNAs, respectively. Functional annotation indicated that the trans-targets were also enriched in a number of reproduction-related processes, including “regulation of hormone levels”, “dynein binding”, “calcium signaling pathway” and “ovarian steroidogenesis” for up-DElncRNAs (Table S5e,f), and “calcium ion homeostasis” for down-DElncRNAs (Table S5g,h). In addition, “MAPK signaling pathway”, “Wnt signaling pathway”, and “TGF-beta signaling pathway” were also enriched among the predicted target genes of DEmiRNAs (Table S6a,b).

### 3.3. DEmRNAs Participate in the Steroid Hormone Biogenesis of Gynogenetic P. olivaceus

Steroid biosynthesis in teleosts is controlled by the hypothalamic–pituitary–interrenal (HPI) and the HPG axis which play essential roles in the regulation of sexual development [37]. Previous transcriptomic studies from normal *P. olivaceus* gonads [17] as well as ovaries of fertile and sterile *P. olivaceus* by mitogynogenesis [22] both revealed differentially expressed genes related to steroid biosynthesis. Here, we focused on the gonadal steroidogenesis pathway with 67 genes identified, in which 18 genes were up-regulated in neo-male testes and 11 genes were up-regulated in ovaries of gynogenetic *P. olivaceus* (Figure 3b,c and Table S7).

In vertebrates, most kinds of steroid hormones are synthesized de novo from the precursor cholesterol [38]. Its transmembrane transport is controlled by *star*, which transfers cholesterol from its intracellular source into the mitochondria [37,38]. *Star* was highly expressed in the neo-male testis (Figure 3b), which was the same in the testis of normal *P. olivaceus* [17]. *Cyp11a* is the only enzyme that removes the side chain of cholesterol resulting in pregnenolone and is therefore the only entrance into the steroidogenesis process [38]. *Cyp11a* expressed as a male-biased gene in the neo-male testis (Figure 3b), while it was not differentially expressed in normal *P. olivaceus* gonads [17] and was down regulated in sterile ovaries to fertile ovaries [22]. *Cyp17a* is responsible for 17,20-lyase and 17-alphahydroxylase activities and is the only enzyme that is responsible for the conversion of C21 steroids (cortisol) to C19 steroids (11-ketotestosterone) [38]. Two *cyp17a*s were identified in *P. olivaceus*, with *cyp17a-I* possessing both hydroxylase and lyase activities (highly expressed in the neo-male testis, Figure 3b), while *cyp17a-II* only having hydroxylase activity (poorly expressed in both ovary and testis) [36]. Neither of the two *cyp17a* genes was differentially expressed in normal *P. olivaceus* gonads [17]. *Cyp19a1* is involved in the synthesis of C18 steroids (estradiol) and is the most essential enzyme in hormonal control of sexual development in teleosts, which was highly expressed in both gynogenetic (Figure 3c) and normal [17] *P. olivaceus* ovaries. Moreover, *hsd3b1* is responsible for the formation of several steroid hormones such as progesterone and testosterone [38], which was highly expressed in the neo-male testis (Figure 3b) but over expressed in ovaries of normal *P. olivaceus* [17]. *Hsd17b1* converts inactive estrone (E1) to active, receptor-binding estradiol (E2), which was highly expressed in both gynogenetic (Figure 3c) and normal [17] *P. olivaceus* ovaries. In addition, other steroidogenesis-related genes, such as *igf1r*, *fshr*, *bmp6* and *arb*, were also highly expressed in neo-male testes, while *bmp15* and *ptgs2*/*COX2* were highly expressed in ovaries (Figure 3c). Overall, there were more highly expressed genes (*cyp11a*, *cyp17a I*, *hsd3b1*, *igf1r*, *bmp6* and *fshr*) in the neo-male testis than in the normal male testis [17], which was presumably necessary for the development of normal sperm in the sex-reversed XX neo-males.

### 3.4. Sperm Motility-Related mRNA–miRNA–lncRNA Interaction in Gynogenetic P. olivaceus

**Figure 4:**
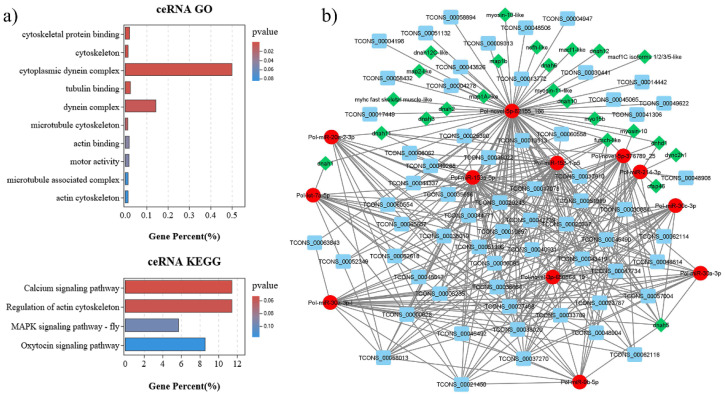
The mRNA–miRNA–lncRNA interaction with sperm motility-related pathways in gynogenetic *P. olivaceus*. (**a**) GO and KEGG enrichment of DEmRNAs from the ceRNA network; (**b**) ceRNA sub-network with cytoskeleton and microtubule-related DEmRNAs. Green diamonds represent DEmRNAs, blue boxes represent DElncRNAs and red circles represent DEmiRNAs.

Transcripts with shared miRNA binding sites may compete for post-transcriptional regulation as ceRNAs [6]. A total of 91 DEmRNAs, 64 DElncRNAs, and 98 DEmiRNAs were identified in the mRNA–miRNA–lncRNA interaction network in gynogenetic *P. olivaceus* gonads (Table S8). To validate their potential function, GO and KEGG annotation of DEmRNAs in the network were performed, which were mostly enriched in “cytoskeleton”, “cytoplasmic dynein complex”, “microtubule cytoskeleton”, “tubulin binding”, and “actin binding” (Figure 4a and Table S9a,b), suggesting their possibly essential function in sperm motility during spermatogenesis. The DEmRNAs involved in these functions, including 4 microtubule-associated proteins (*maps*), 5 *myosins* and 11 dynein axonemal heavy chains (*dnahs*), were selected for the subnetwork construction, which was composed of 12 DEmiRNA nodes, 24 DEmRNA nodes and 63 DElncRNA nodes (Figure 4b). For example, in this subnetwork, the ovary-biased *let-7* was the hub miRNA to interact with *dnah1* and 18 DElncRNAs, and the testis-biased *miR-20a* was the hub miRNA to interact with *dnah11* and 14 DElncRNAs, while the novel *miRNA-82155_166* could collaborate with most of the 22 DEmRNAs and 57 DElncRNAs (Figure 4b).

During spermatogenesis, the round spermatids differentiate into spermatozoa in which significant changes are present in the cytoskeletal structures, and microtubules play vital roles in these orderly processes [39,40]. Microtubules function through collaboration with motor proteins such as dynein and kinesin, which move in different directions along the microtubules [41]. Among these motor proteins, dynein is mainly involved in the intracellular transport of flagella [41]. *Dnah* genes, which encode inner dynein heavy chains that have microtubule motor activity and ATPase activity, are responsible for sperm flagellar assembly and sperm motility [42,43]. Mutations in *dnah* genes were characterized in patients affected by isolated asthenozoospermia [44] and also contributed to male infertility and reduced ciliary beat frequency in mice [45]. In this study, we obtained increased expression of 11 *dnah* genes in the neo-male testis that putatively interact with lncRNAs and miRNAs (Figure 4b), which may be associated with the promotion of sperm motility in sex-reversed neo-male *P. olivaceus*. Moreover, myosins also play essential roles in spindle assembly and positioning, acrosomal formation and spermatid differentiation during spermatogenesis [39]. In the network, 5 *myosin* genes were highly expressed in the neo-male testes (Figure 4b), including *myosin 10* and *10 like*, myosin *11*, *myosin 15b* and *myosin heavy chain C*, suggesting their essential functions in neo-male spermatogenesis. The similar expression patterns of these sperm motility-related genes were also reported in other teleosts such as in the large yellow croaker (*Larimichthys crocea*) testis [46], as well as in the diploid and tetraploid common carp (*Cyprinus carpio* L.) testis [42].

### 3.5. Clustered miRNAs and miRNA Host lncRNAs in Gynogenetic P. olivaceus

In general, miRNAs are often distributed in clusters which are evolutionarily conserved and may co-regulate functionally related genes or pathways [47]. We defined miRNA clusters consisting of at least two miRNAs, with a maximum distance of 10 kb across the genome. In the *P. olivaceus* genome, 106 miRNA clusters were identified with 427 miRNAs accounting for 51.8% of all miRNAs, which was more than in other selected teleosts, except *G. aculeatus* (Table 2). Moreover, a subclass of lncRNAs could be transcribed from miRNA host genes (MIRHGs) as pri- or pre-miRNA precursors and are classified as miRNA host gene lncRNAs (lncmiRHGs). We therefore provided evolutionary trajectories of the largest miRNA clusters identified in the *P. olivaceus* genome.

**Figure 5:**
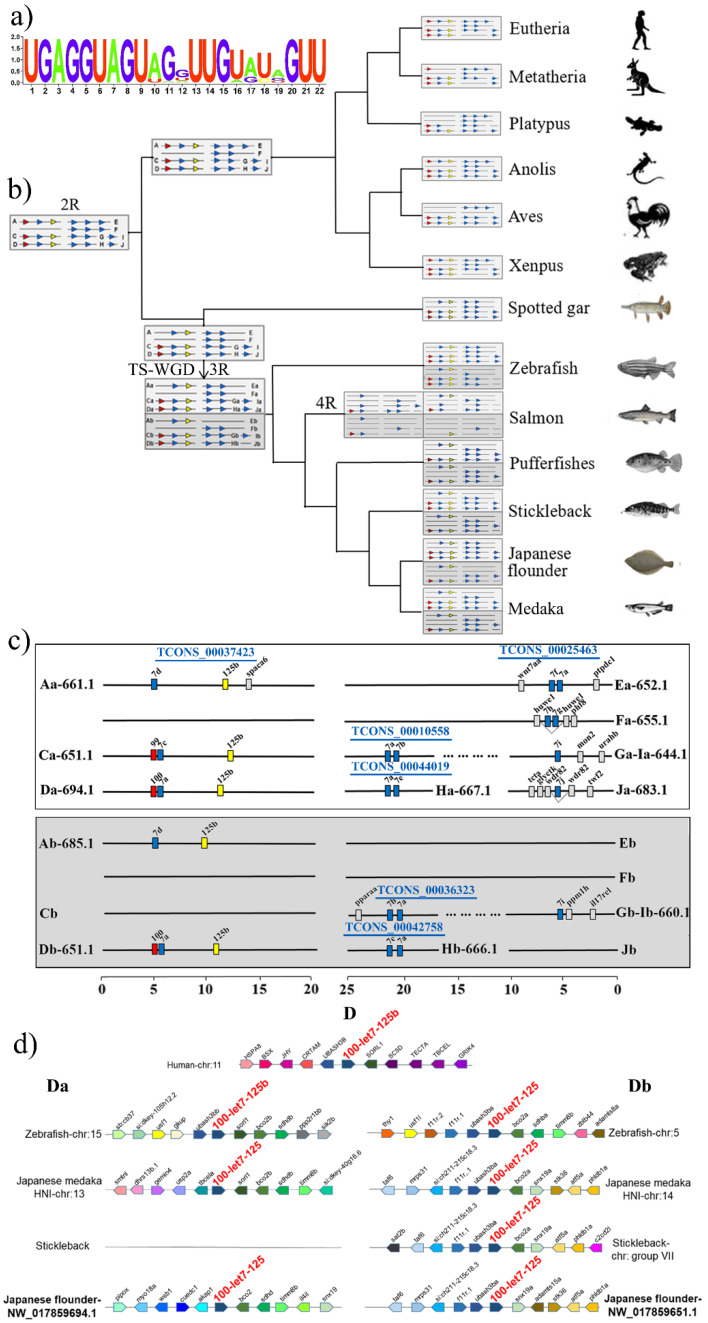
Characterization of *let-7* clusters in *P. olivaceus* genome. (**a**) Consensus sequence of the 20 *P. olivaceus let-7* members with seed sequence “GAGGUAG”; (**b**) Putative evolutionary trajectory of the *let-7*/*miR-125b* clusters across vertebrates (modified and referenced from [48]). Red triangles represent *miR-100*, yellow triangles represent *miR-125b*, while *let-7* sequences are shown as blue triangles. 1R/2R indicate two rounds of whole genome duplications, while TS-WGD represents the additional teleost-specific genome duplication. The duplicate clusters in teleost are highlighted as two boxes; (**c**) Detailed annotation of *P. olivaceus let-7* clusters with six putative lncRNA precursors; (**d**) Synteny for *let-7* cluster D. The pentagon’s direction shows the gene direction compared with the reference gene.

The most frequently clustered miRNAs in *P. olivaceus* genome were the *let-7* family. Vertebrate genomes normally possess a number of *let-7* paralogs, some clustered in polycistronic transcripts with *miR-125b* and *miR-99/100*, and some located in isolation [48,49]. Our survey resulted in 20 *P. olivaceus let-7* copies, with “GAGGUAG” as the seed region for all of them (Figure 5a), suggesting their high conservation across species in sequence and function. The arrangement of *let-7* paralogs was also well conserved, with 17 *let-7* members distributed into 11 clusters, while 3 *let-7*s were isolated, which was similar to other teleosts (Figure 5b). Specifically, there were three mixed clusters A, C and D (with *miR-100* and *miR-125b*), four homogeneous clusters E, F, G and H (duplicated *let-7*s), and two isolated loci I and J (single *let-7*) being traced throughout all vertebrates (Figure 5b,c). Orthology of these clusters was established based on synteny analysis (Figure 5d and Figure S1). The teleost-specific whole genome duplication (TS-WGD) contributed to the *let-7* divergence among teleost lineages. For example, the teleost Da and Db shared neighboring protein-coding genes with the human D locus, e.g., *ubash3* and *sorl1* (Figure 5d). There were also changes that can be attributed to the teleost specific loss of A-*miR-100* and E-*let-7-3* (Figure 5b,c). Moreover, loci I and J were in a weak association with the G and H clusters, respectively. The G and I loci were located at the same scaffold (Figure 5c), although separated by many megabases, which was similar with that in tetrapods [49,50]. The ancestral *let-7* clusters were normally tightly linked as a polycistronic primary transcript [49]. Although the order of miRNAs from each cluster is conserved among vertebrates, the distances in mixed clusters (A, C and D) substantially increased in teleosts, namely A-*let-7*/A-*miR-125b*, C-*let-7*/C-*miR-125b* and D-*let-7*/D-*miR-125b* (Figure 5c), with *miR-125b* being many kilobases away, indicating unlikely polycistronic *let-7*/*miR125b* transcription in *P. olivaceus*. Moreover, all homogeneous *let-7* clusters (E, F, G and H) appeared to be closely related with each other, suggesting their origin through duplication (Figure 5c).

**Figure 6:**
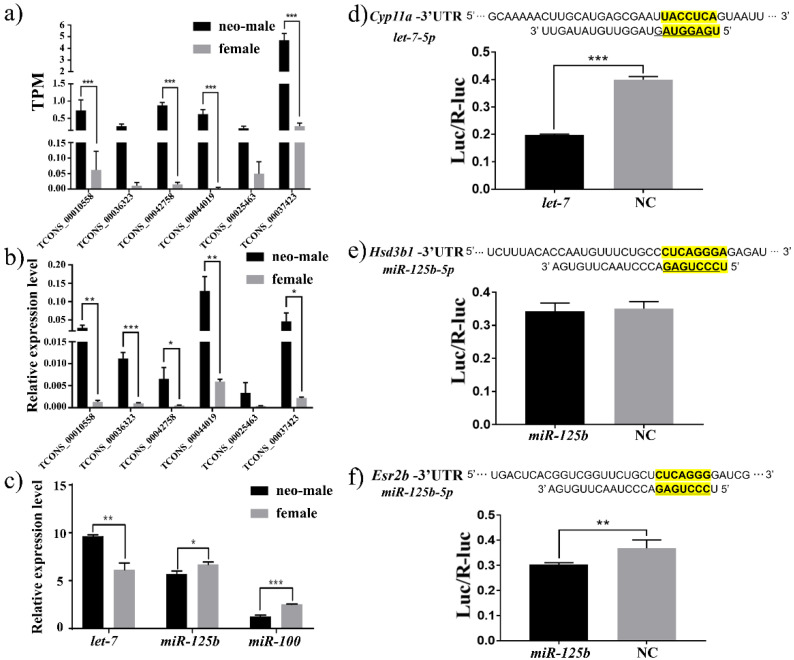
Functional investigation of *let-7*/*miR-125b* cluster in gynogenetic *P. olivaceus*. Expression of six putative lncRNAs as *let-7* precursors by (**a**) RNA-seq and (**b**) qRT-PCR; (**c**) expression of *let-7*, *miR-125b* and *miR-100* by qRT-PCR; dual-luciferase reporter analysis of *let-7* targeting *cyp11a* (**d**) and *miR-125b* targeting *hsd3b1* (**e**) and *esr2b* (**f**), with the bold yellow regions as the complementary target sites. Data are shown as mean ± SD (*n* = 3). Asterisks indicate statistical significance between groups (* *p* < 0.05; ** *p* < 0.01; *** *p* < 0.001).

In some cases, the clusters were contained in non-coding primary precursors (5 lncRNAs covering Aa, Ea, Ha, Gb and Hb), while in other cases, an antisense transcript could be found (TCONS_00010558 for Ga) (Figure 5c). The six lncRNAs covering *let-7* or *miR-125b* represented male-biased expression (Figure 6a,b and Table S7), which was not entirely consistent with the expression of mature miRNAs (Figure 6c and Figure S2), indicating the difference between putative pri-miRNA transcripts (lncRNAs) and mature miRNAs (*let-7*s) after micro-processing. For example, both neighboring *miR-100* and *miR-125b* were female-biased in cluster A, C and D, while the middle *let-7*s were not differentially expressed (Figure S2), suggesting uneven pri-miRNA processing or non-polycistronic transcription. Other duplicated or singleton *let-7*s represent diverse expression patterns, with both male-biased (Ea, Fa, Ia and Ja) and female-biased (Ga and Gb) expression (Figure 6c and Figure S2), indicating potential functional divergence of *let-7* clusters.

### 3.6. Regulation of Steroidogenesis Pathway by let-7/miR-125b in P. olivaceus

To investigate the potential function of *let-7*/*miR-125b* clusters in sexual development, we searched for miRNA targets in the 3′-UTR of *P. olivaceus* protein-coding genes using TargetScan and miRanda. In total, 7,690 target genes were detected corresponding to functions in “hormone-mediated signaling pathway”, “response to hormone”, “oocyte meiosis”, “GnRH signaling”, and “estrogen signaling” et al. by GO and KEGG classification (Table S10a,b), in which known genes were found involved in sexual development as putative targets of *let-7* (*sox2*, *fst*, *cyp11a*, *star*, *wnt9a*, *err2,* et al.) and *miR-125b* (*hsd3b1* and *esr2b*). To confirm the *let-7* regulation of *cyp11a* and *miR-125b* targeting of *hsd3b1* and *esr2b*—all genes from the steroid biogenesis pathway—we generated dual-luciferase reporters fused with *cyp11a*, *hsd3b1* and *esr2b* 3′-UTR containing the target sites, respectively (Figure 6d–f). Dual-luciferase assays showed that *let-7* significantly reduced the activity of *cyp11a* reporter, and *miR-125b* significantly repressed the activity of *esr2b* reporter, but did not repress the *hsd3b1* reporter (Figure 6d–f and Table S11), suggesting the regulatory role of *let-7*/*miR-125b* in steroidogenesis via *cyp11a* and *esr2b*. *Cyp11a* is important for the production of all three major steroid hormones, mineralocorticoids, glucocorticoids, and sex steroids [51], and many biological effects associated with estrogen receptors can be linked to the regulation of estrogen-regulated miRNAs [52]. There was also evidence of miRNA-mediated regulation of testicular and ovarian steroidogenesis, with both *let-7* or *miR-125b* targeting *star*, *cyp11b2*, *cyp17a1*, *creb1* and *sr-b1* et al. in model organisms [53,54], while their targeting *cyp11a* and *esr2b* was only specific in *P. olivaceus* but not in other teleosts (data not shown), therefore indicating possible diverse target but conserved function of *let-7*/*miR-125b* clusters in steroid homeostasis. Further regulation investigation may warrant more detailed functions of certain specific *let-7* clusters in teleost steroidogenesis, especially for the sex-reversed neo-male *P. olivaceus*.

## 4. Conclusions

A comprehensive transcriptome analysis including mRNAs, lncRNAs and miRNAs was performed in the gonads of gynogenetic *P. olivaceus*, which revealed that ncRNAs, such as miRNAs and lncRNAs, were actively expressed in the germline. A considerable amount of steroid biogenesis and sperm motility-related genes and pathways were identified in gynogenetic *P. olivaceus*, which may interact with *let-7*/*miR-125b* clusters and precursor lncRNAs and have significant functions during spermatogenesis of the neo-male *P. olivaceus*. Highly conserved mRNAs and ncRNAs could potentially interact with each other, which will enrich the genomic information for *P. olivaceus* and pave the way towards the understanding of molecular mechanisms underlying the gynogenesis effect and sex reversal in sexual development and gametogenesis of *P. olivaceus*.

## Figures and Tables

**Table 1:** Differentially expressed coding and non-coding RNAs in gynogenetic *P. olivaceus* gonads. Percentage in parenthesis represents the proportion of regulated RNAs to their total number.

DE RNAs	mRNAs		lncRNAs		miRNAs	
	Up	Down	Up	Down	Up	Down
neo-male vs. female	3541 (15.5%)	3231 (14.1%)	1870 (22.5%)	414 (5.0%)	146 (17.7%)	98 (11.9%)

**Table 2:** The percentage of clustered miRNAs out of the total miRNAs among teleost genomes.

Species	miRNAs	miRNA Clusters	miRNAs in Clusters	miRNAs in Clusters/miRNAs
*P. olivaceus*	824	106	427	52%
*N. furzeri*	754	83	213	28%
*D. rerio*	765	96	305	40%
*O. latipes*	366	58	151	41%
*G. aculeatus*	504	68	299	59%
*T. rubripes*	337	59	143	42%

## Data Availability

The datasets generated for this study can be found in the NCBI Sequence Read Archive (SRA) BioProject PRJNA764760.

## Supplementary Materials

The following supporting information can be downloaded at: https://www.mdpi.com/article/10.3390/biology11020213/s1, Table S1: Primers used for (a) qRT-PCR analysis and (b) luciferase reporter vector construction; Table S2: Overview of the (a) RNA-seq and (b) small RNA-seq for each sample; Table S3: TPM values for (a) mRNAs, (b) lncRNAs, and (c) miRNAs from sequencing analysis; Table S4: GO and KEGG classification of up- and down- DEmRNAs; Table S5: GO and KEGG classification of mRNAs colocalized (cis-) and co-expressed (trans-) with DElncRNAs; Table S6: GO and KEGG classification of predicated targets of DEmiRNAs; Table S7: qRT-PCR analysis for selected DEmRNAs, DElncRNAs and DEmiRNAs; Table S8: mRNA–miRNA–lncRNA network analysis; Table S9: GO and KEGG classification of ceRNAs; Table S10: GO and KEGG classification of predicated targets of let-7; Table S11: Luciferase activity for dual-luciferase reporter assay; Figure S1: Synteny analysis of let-7 clusters in vertebrates. The pentagon’s direction indicates the gene direction compared with the reference gene; Figure S2: Differential expression of let-7 cluster members from small RNA-seq analysis.
