# Supplementary material for: Transcriptomic Analysis Reveals Functional Interaction of mRNA–lncRNA–miRNA in Steroidogenesis and Spermatogenesis of Gynogenetic Japanese Flounder (Paralichthys olivaceus)
Source: Biology (Basel). 2022 Jan 28;11(2):213. doi: 10.3390/biology11020213 (PMC8869744; doi:10.3390/biology11020213)
Supplement: Supplementary file 1 [file biology-11-00213-s001.zip › Figure S1.let7 synteny.pdf]

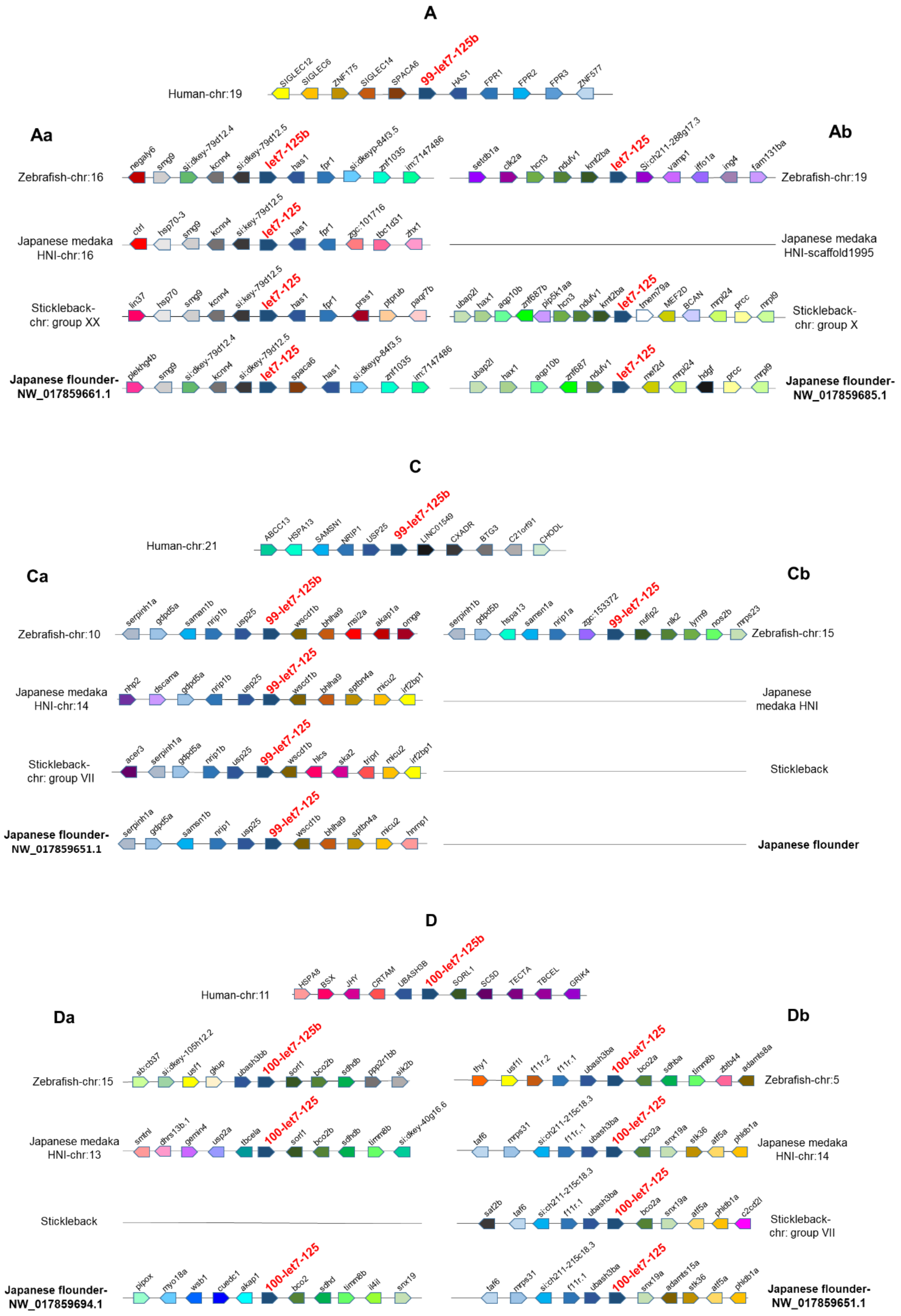

A
Human-chr:19
SIGLEC12
SIGLEC6
ZNF175
SIGLEC14
SPACA6
99-let7-125b
HAS1
FPR1
FPR2
FPR3
ZNF577
Aa
Ab
Zebrafish-chr:16
negaly6
smg9
si:dkey-79d12.4
kcnn4
si:dkey-79d12.5
let7-125b
has1
fpr1
si:dkeyp-84f3.5
znf1035
im:7147486
setdb1a
clk2a
hcn3
ndufv1
kmt2ba
let7-125
Si:ch211-288g17.3
vamp1
ifo1a
ing4
fam131ba
Zebrafish-chr:19
Japanese medaka HNI-chr:16
ctrl
hsp70-3
smg9
kcnn4
si:key-79d12.5
let7-125
has1
fpr1
zgc:101716
tbc1d31
zhx1
Japanese medaka HNI-scaffold1995
Stickleback-chr: group XX
lin37
hsp70
smg9
kcnn4
si:key-79d12.5
let7-125
has1
fpr1
prss1
ptpnub
pagr7b
ubap2l
hax1
aqp10b
znf687b
pip5k1aa
hcn3
ndufv1
kmt2ba
let7-125
tmem79a
MEF2D
BCAN
mrpl24
prcc
mrpl9
Stickleback-chr: group X
Japanese flounder-NW_017859661.1
plekhg4b
smg9
si:dkey-79d12.4
kcnn4
si:dkey-79d12.5
let7-125
spaca6
has1
si:dkeyp-84f3.5
znf1035
im:7147486
ubap2l
hax1
aqp10b
znf687
ndufv1
let7-125
mef2d
mrpl24
hdgf
prcc
mrpl9
Japanese flounder-NW_017859685.1
C
Human-chr:21
ABCC13
HSPA13
SAMSN1
NRIP1
USP25
99-let7-125b
LINC01549
CXADR
BTG3
C21orf91
CHODL
Ca
Cb
Zebrafish-chr:10
serpinh1a
gdpd5a
samsn1b
nrip1b
usp25
99-let7-125b
wscd1b
bhlha9
msi2a
akap1a
omga
serpinh1b
gdpd5b
hspa13
samsn1a
nrip1a
zgc:153372
99-let7-125
nufip2
nlk2
tymm9
nos2b
mrps23
Zebrafish-chr:15
Japanese medaka HNI-chr:14
nhp2
dscama
gdpd5a
nrip1b
usp25
99-let7-125
wscd1b
bhlha9
spbn4a
micu2
irf2bp1
Japanese medaka HNI
Stickleback-chr: group VII
acer3
serpinh1a
gdpd5a
nrip1b
usp25
99-let7-125
wscd1b
hlcs
ska2
tripl
micu2
irf2bp1
Stickleback
Japanese flounder-NW_017859651.1
serpinh1a
gdpd5a
samsn1b
nrip1
usp25
99-let7-125
wscd1b
bhlha9
sptbn4a
micu2
hnrnp1
Japanese flounder
D
Human-chr:11
HSPA8
BSX
JHY
CRTAM
UBASH3B
100-let7-125b
SORL1
SC5D
TECTA
TBCEL
GRIK4
Da
Db
Zebrafish-chr:15
sb:cb37
si:dkey-105h12.2
usf1
gkup
ubash3bb
100-let7-125b
sorl
bco2b
sdhdb
ppp2r1bb
sik2b
thy1
usf1l
f11r.2
f11r.1
ubash3ba
100-let7-125
bco2a
sdhba
timm8b
zbtb44
adamts8a
Zebrafish-chr:5
Japanese medaka HNI-chr:13
smtnl
dhrs13b.1
gemin4
usp2a
tbcela
100-let7-125
sorl
bco2b
sdhdb
timm8b
si:dkey-40g16.6
taf6
mrps31
si:ch211-215c18.3
f11r.1
ubash3ba
100-let7-125
bco2a
snx19a
stk36
atf5a
phldb1a
Japanese medaka HNI-chr:14
Stickleback
sat2b
taf6
si:ch211-215c18.3
f11r.1
ubash3ba
100-let7-125
bco2a
snx19a
atf5a
phldb1a
c2cd2l
Stickleback-chr: group VII
Japanese flounder-NW_017859694.1
pipox
myo18a
wsb1
cuedc1
akap1
100-let7-125
bco2
sdhd
timm8b
il4il
snx19
taf6
mrps31
si:ch211-215c18.3
f11r.1
ubash3ba
100-let7-125
snx19a
adamts15a
stk36
atf5a
phldb1a
Japanese flounder-NW_017859651.1

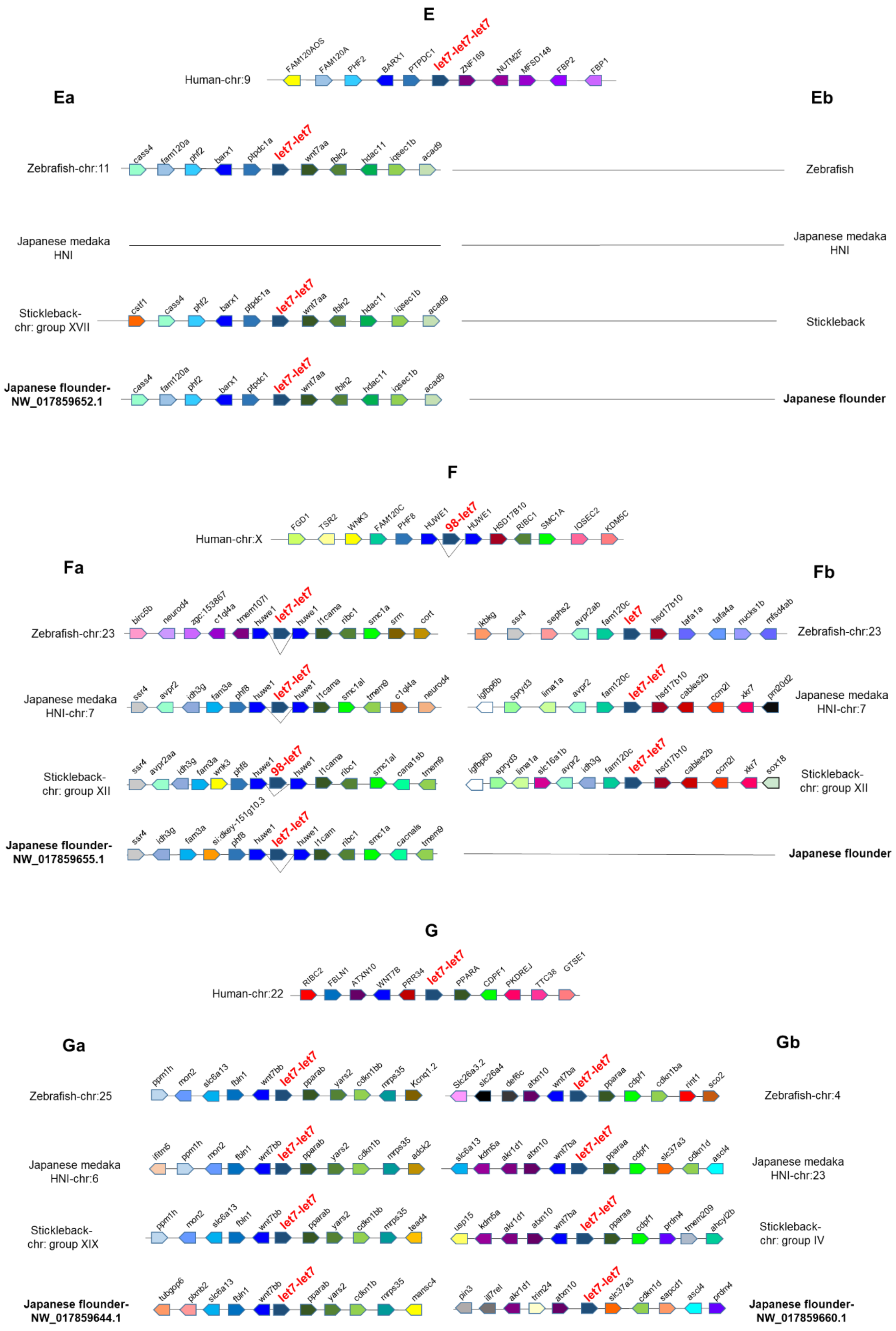
E
Ea
Eb
Human-chr:9
FAM120AOS FAM120A PHF2 BARX1 PTPDC1 let7-let7-let7 ZNF169 NUTM2F MFSD14B FBP2 FBP1
Zebrafish-chr:11
cass4 fam120a phf2 barx1 ptpdc1a let7-let7 wnt7aa fbln2 hdac11 iqsec1b acad9
Zebrafish
Japanese medaka HNI
Japanese medaka HNI
Stickleback-chr: group XVII
cstf1 cass4 phf2 barx1 ptpdc1a let7-let7 wnt7aa fbln2 hdac11 iqsec1b acad9
Stickleback
Japanese flounder-NW_017859652.1
cass4 fam120a phf2 barx1 ptpdc1 let7-let7 wnt7aa fbln2 hdac11 iqsec1b acad9
Japanese flounder
F
Human-chr:X
FGD1 TSR2 WNK3 FAM120C PHF8 HUWE1 98-let7 HUWE1 HSD17B10 RIBC1 SMC1A IQSEC2 KDM5C
Fa
Fb
Zebrafish-chr:23
birc5b neurod4 zgc:153867 c1ql4a tmem107l huwe1 let7-let7 huwe1 l1cama ribc1 smc1a srm cort
ikbkg ssr4 sephs2 avpr2ab fam120c let7 hsd17b10 tafa1a tafa4a nucks1b mfsd4ab
Zebrafish-chr:23
Japanese medaka HNI-chr:7
ssr4 avpr2 idh3g fam3a phf8 huwe1 let7-let7 huwe1 l1cama smc1al tmem9 c1ql4a neurod4
igfbp6b spryd3 lima1a avpr2 fam120c let7-let7 hsd17b10 cables2b ccm2l xkr7 prr20d2
Japanese medaka HNI-chr:7
Stickleback-chr: group XII
ssr4 avpr2aa idh3g fam3a wnk3 phf8 huwe1 98-let7 huwe1 l1cama ribc1 smc1al cana1sb tmem9
igfbp6b spryd3 lima1a slc16a1b avpr2 idh3g fam120c let7-let7 hsd17b10 cables2b ccm2l xkr7 sox18
Stickleback-chr: group XII
Japanese flounder-NW_017859655.1
ssr4 idh3g fam3a si:dkey-151g10.3 phf8 huwe1 let7-let7 huwe1 l1cam ribc1 smc1a cacnals tmem9
Japanese flounder
G
Human-chr:22
RIBC2 FBLN1 ATXN10 WNT7B PRR34 let7-let7 PPARA CDPF1 PKDREJ TTC38 GTSE1
Ga
Gb
Zebrafish-chr:25
ppm1h mon2 slc6a13 fbln1 wnt7bb let7-let7 pparab yars2 cdkn1bb mrps35 Kcnq1.2
Slc26a3.2 slc26a4 def6c atxn10 wnt7ba let7-let7 pparaa cdpf1 cdkn1ba rint1 sco2
Zebrafish-chr:4
Japanese medaka HNI-chr:6
ifitm5 ppm1h mon2 fbln1 wnt7bb let7-let7 pparab yars2 cdkn1b mrps35 adck2
slc6a13 kdm5a akr1d1 atxn10 wnt7ba let7-let7 pparaa cdpf1 slc37a3 cdkn1d ascl4
Japanese medaka HNI-chr:23
Stickleback-chr: group XIX
ppm1h mon2 slc6a13 fbln1 wnt7bb let7-let7 pparab yars2 cdkn1bb mrps35 tead4
usp15 kdm5a akr1d1 atxn10 wnt7ba let7-let7 pparaa cdpf1 prdm4 tmem209 ahcyl2b
Stickleback-chr: group IV
Japanese flounder-NW_017859644.1
tubgcp6 phmb2 slc6a13 fbln1 wnt7bb let7-let7 pparab yars2 cdkn1b mrps35 mansc4
pin3 lif7rel akr1d1 trim24 atxn10 let7-let7 slc37a3 cdkn1d sapcd1 ascl4 prdm4
Japanese flounder-NW_017859660.1

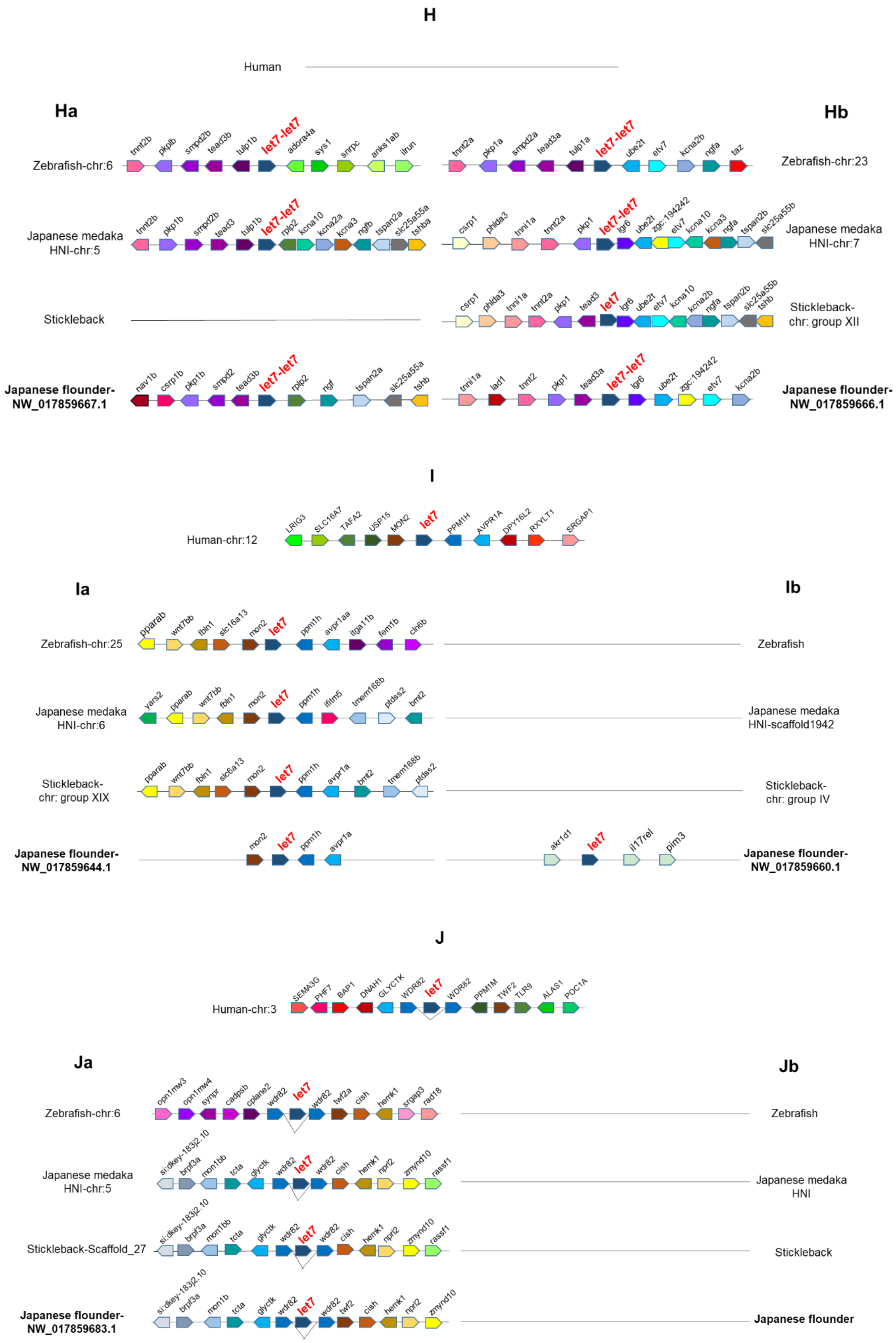

Figure S1. Synteny analysis of let-7 clusters in vertebrates. The pentagon's direction indicates the gene direction compared with the reference gene.
