# Supplementary material for: Transcriptomic Analysis Reveals Functional Interaction of mRNA–lncRNA–miRNA in Steroidogenesis and Spermatogenesis of Gynogenetic Japanese Flounder (Paralichthys olivaceus)
Source: Biology (Basel). 2022 Jan 28;11(2):213. doi: 10.3390/biology11020213 (PMC8869744; doi:10.3390/biology11020213)
Supplement: Supplementary file 1 [file biology-11-00213-s001.zip › Table S1ab.Primers for qRT-PCR and vector.pdf]

Table S1. Primers used for (a) qRT-PCR analysis and (b) luciferase reporter vector construction.

(a) Primers used for qRT-PCR analysis.

Primer	Sequence 5'-3'
star2-Fw	TGGTGTGGACGAACTCTATG
star2-Rv	GGAAATCCCTTTGGCCAATTA
cyp11a-Fw	CCGTCTACTGAAACTGGTTGG
cyp11a-Rv	GGAGAGCTTGTCCAGCATTAG
cyp17a1-Fw	GAAACCACAACCACTGTACTC
cyp17a1-Rv	GATCCGTAACACCTCCCTTAT
hsd3b1-Fw	ATGATGATCCGACCTCTCATAC
hsd3b1-Rv	CAGTTCTGTGGCTAACCATTC
hsd17b7-Fw	GTCAAGTACCACAGCTTAACATC
hsd17b7-Rv	TCCTTCAGTTCCTTCCTTACTT
hsd17b12a-Fw	GCTGGAGAATTGCACTCTTTAC
hsd17b12a-Rv	CCATCAGCAGGGTAAATGAAAC
let-7(644.1)-Fw	GATCAGCTCGCAACCAATAC
let-7(644.1)-Rv	CACCTTGTTAGCCCTCTATCT
let-7(660.1)-Fw	TCCAATGTATCCCTCCTCTC
let-7(660.1)-Rv	CAGATGGCGCCAGATAATAC
let-7(666.1)-Fw	CTAGTGGAGTCACAGGAGATAG
let-7(666.1)-Rv	CTGACCAATCACACAGAGAAG
let-7(667.1)-Fw	AGGAAGCTGTCTTGGATATTG
let-7(667.1)-Rv	GAGGGCTGAAGAGGAAATAAG
let-7(652.1)-Fw	GTCTGTGGTGTGGAAGAAATA
let-7(652.1)-Rv	CCTATGAGGCCTGTGAATATG
miR-125(661.1)-Fw	CCTTGGCTGTCTTCCTATTG
miR-125(661.1)-Rv	TAAACCACTGATGCCTGTTAG
β-actin-Fw	CCACCGCAAATGCTTCTA
β-actin-Rv	ACTGTCTCCATCGTTCCA
UBCE-Fw	TTACTGTCCATTTCCCCACTGAC
UBCE-Rv	GACCACTGCGACCTCAAGATG
let-7	TGAGGTAGTAGGTTGTATAGTT
miR-125b	TCCCTGAGACCCTAACTTGTGA
miR-100	AACCCGTAGATCCGATCTTGTG
miR-22-3p	AAGCTGCCAGCTGAAGAACTGT
miR-23a-3p	ATCACATTGCCAGGGATTTCCA

The reverse primer used for miRNA detection was universal downstream primer (mRQ 3' Primer, Takara).

(b) Primers used for luciferase reporter vector construction.

Primer	Sequence 5'-3'
Cyp11a-3'UTR-Fw(XhoI)	CCGCTCGAGCAGATTCTTACATCAGCAAAGCAC

Cyp11a-3'UTR-Rv(SalI)	ACGCGTCGACGTCCAGAGCAGTATCAGCCGT
Hsd3b1-3'UTR-Fw(XhoI)	CCGCTCGAGGAATGGTTAGCCACAGAACTGC
Hsd3b1-3'UTR-Rv(SalI)	ACGCGTCGACCCCAGGAAAATAGGTCAAGGA
Esr2b-3'UTR-Fw(XhoI)	CCGCTCGAGCGTAAAGCCAGAGAGTTACAG
Esr2b-3'UTR-Rv(SalI)	ACGCGTCGACGTGTCAGTGGAGTCAAGAAG
