# Supplementary figures and images for: Transcriptomic Analysis Reveals Functional Interaction of mRNA–lncRNA–miRNA in Steroidogenesis and Spermatogenesis of Gynogenetic Japanese Flounder (Paralichthys olivaceus)
Source: Biology (Basel). 2022 Jan 28;11(2):213. doi: 10.3390/biology11020213 (PMC8869744; doi:10.3390/biology11020213)

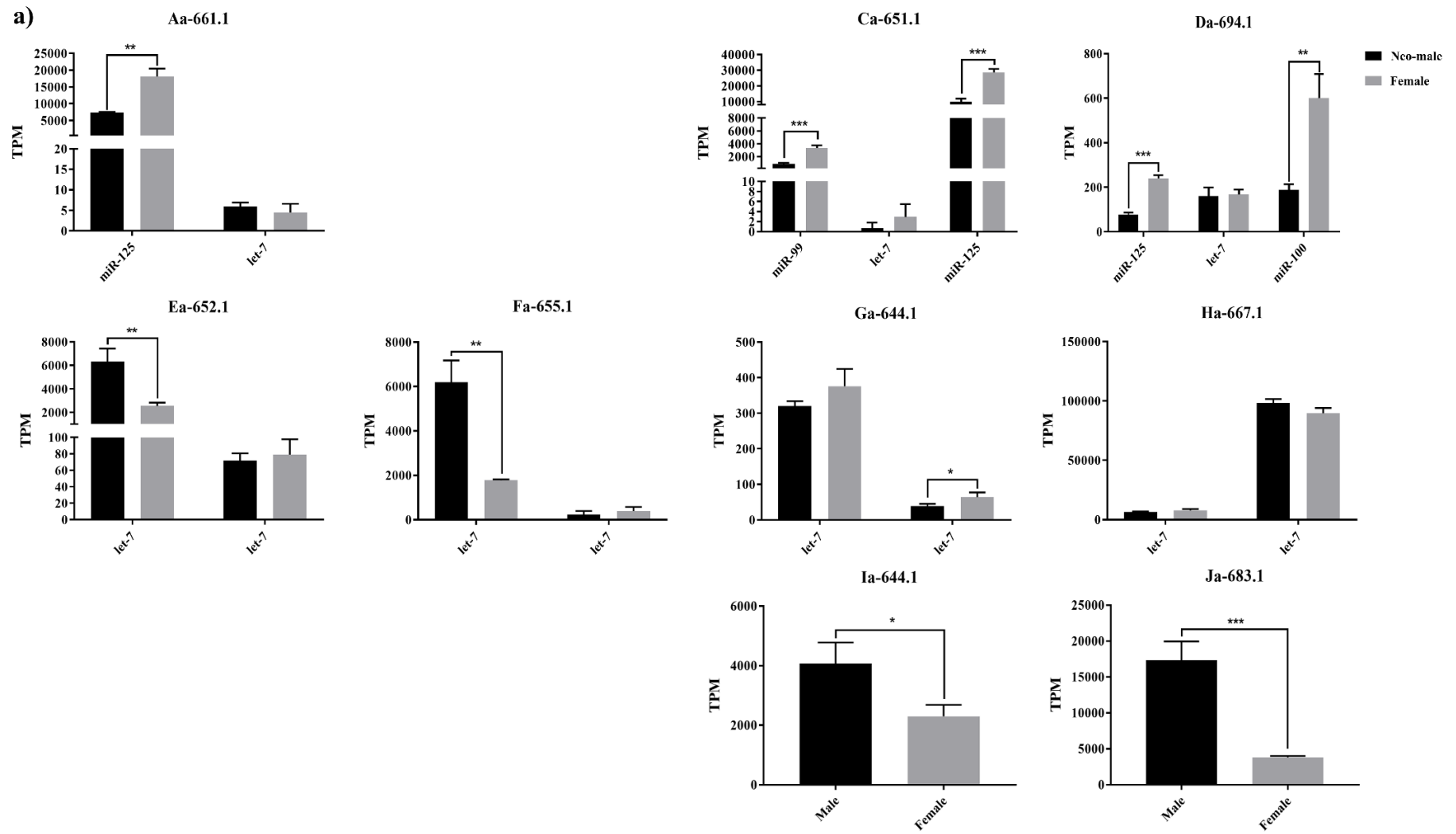

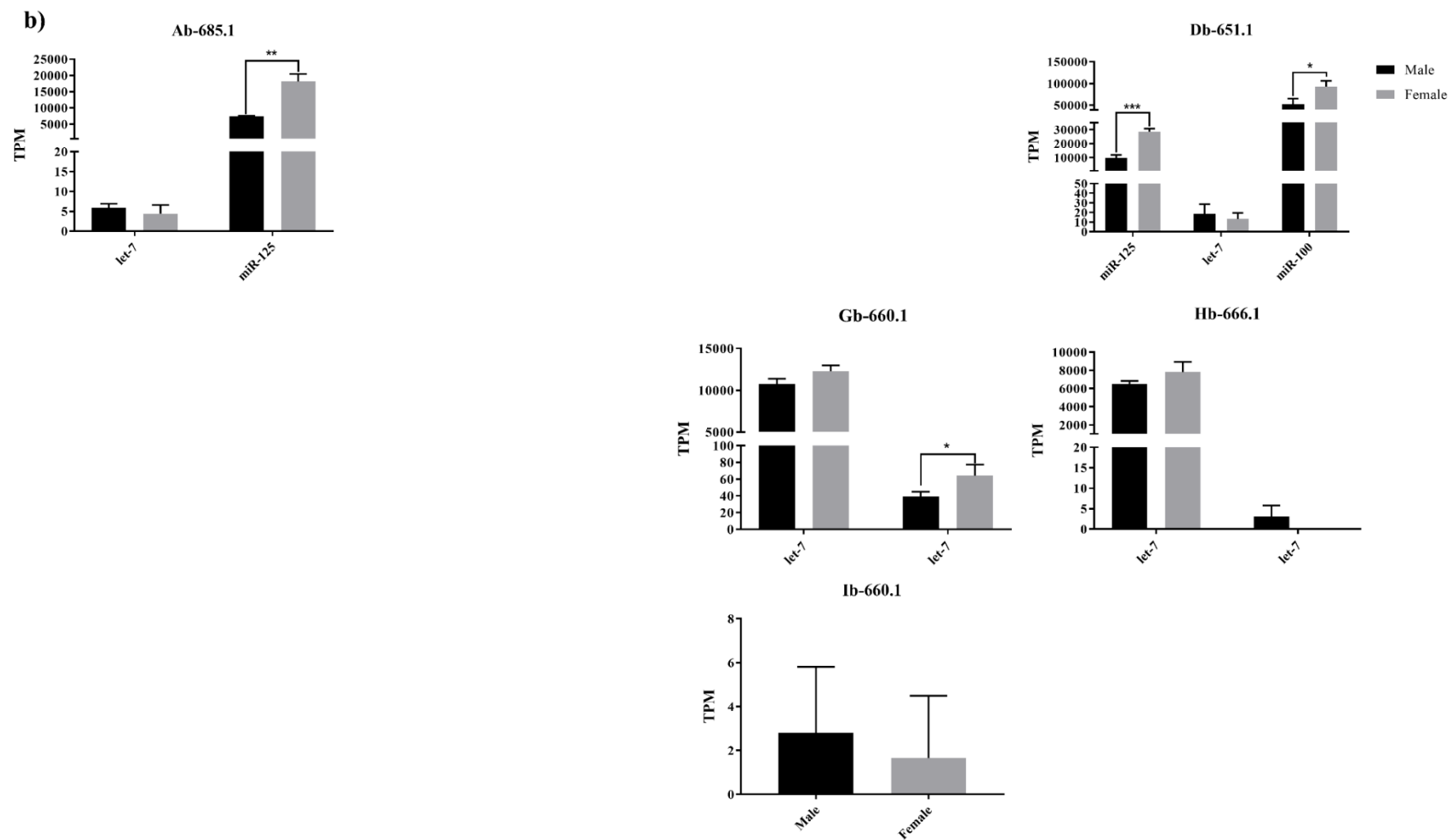

Figure S2. Differential expression of let-7 cluster members from RNA-seq.

Supplement: Supplementary file 1 [file biology-11-00213-s001.zip › Figure S2.let7 cluster expression.pdf]
